# Phenolic content, antioxidant, cytotoxic and antiproliferative effects of fractions of *Vigna subterraenea* (L.) verdc from Mpumalanga, South Africa

**DOI:** 10.1016/j.heliyon.2021.e08397

**Published:** 2021-11-14

**Authors:** Jane N.C. Okafor, Fanie Rautenbauch, Mervin Meyer, Marilize Le Roes-Hill, Taahir Harris, Victoria A. Jideani

**Affiliations:** a Food Science and Technology Department, Cape Peninsula University of Technology, Bellville, 7535, South Africa; b Applied Microbial and Health Biotechnology Institute, Cape Peninsula University of Technology, Bellville, 7535, South Africa; c Nanotechnology Innovation Centre, Department of Biotechnology, University of the Western Cape, Private Bag x17, Bellville, 7535, Cape Town, South Africa; d Biocatalysis and Technical Biology Research Group, Institute of Biomedical and Microbial Biotechnology, Cape Peninsula University of Technology, Bellville, 7535, South Africa; e Nutrition and Toxicology Division, Federal Institute of Industrial Research, Oshodi (FIIRO), P.M.B 21023, Ikeja, Lagos, Nigeria

**Keywords:** Bambara groundnut, Cotyledon, Seed coat, Phenolic, Antioxidant activity, Colon cancer, Cytotoxicity, Antiproliferation

## Abstract

Consistent intake of legumes has been correlated with decreased possibility of developing colorectal cancer (CRC) due to the content of some phytochemicals like polyphenols. Bambara groundnut (BGN) is an underutilized crop with a rich nutritional profile, but have not been exploited for its nutraceutical and medicinal benefits. In this study, total polyphenol, flavonoid (flavonol and flavanol) content, antioxidant activity and cytotoxicity/antiproliferative properties of 70% ethanolic extracts of whole BGN, cotyledon and seed coat on Caco-2 and HT-29 colon cancer cells were evaluated. Seed coat had a significantly (p < 0.05) higher composition of total polyphenol, flavonol and flavan-3-ol (flavanol) compared to whole seed and cotyledon. Antioxidant activity determined with ferric reducing antioxidant power (FRAP), 2,2- azino-bis (3-ethylbenzothiazoline-6-sulfonic acid) (ABTS) and oxygen radical absorbance capacity (ORAC) assays, showed that seed coat with higher polyphenolic content had significantly (p < 0.05) greater antioxidant activity. BGN fractions demonstrated cytotoxic and antiproliferative effects against HT-29 and Caco-2 colon cancer cells in a dose-dependent manner, with seed coat and whole seed exhibiting greater cytotoxicity and higher antiproliferative activity and colon cancer cell inhibition. Extracts of the cotyledon also showed cytotoxic activity and hindered cancer cell growth/division but to a significantly (p < 0.05) lower magnitude. BGN parts indicated a greater cytotoxic effect and potential to slow down Caco-2 colon cancer cell growth and division over HT-29. This result provides new knowledge on the possible health benefits of BGN, as well as the potential for product development and may influence its consumption and utilisation.

## 1 Introduction

Worldwide, consumer's attractions, longing and request for natural components from food is on the rise because of the increased consciousness of diet-related health problems and the important role food play in preventing disease, health promotion and wellness. This is particularly the case in this era of COVID-19 pandemic when consumers are changing their eating behavior by looking out for and embracing/consuming healthier foods and nutraceuticals that could boost and support the immune system, protect them against the lower respiratory tract viruses, and limit or keep under control the spread of SARS-CoV-2 (COVID-19 disease-causing novel coronavirus) (Galanakis, 2020). With the consistent daily increase in Covid-19 infection cases and death, it becomes very important that the scientific community/researchers and the food industry develop fast innovations in the production of functional foods fortified with antioxidants and bioactive components that will not only promote health but will boost the immune system against infection. Also the development of sustainable and modern food systems that will enhance food security, safety and improved nutrition during the post lockdown era (Covid-19 pandemic) is crucial**.**

Legumes contain many phytochemicals with bioactive components that have health-promoting effects. Consequently, legumes/legume-based foods are attracting more research awareness and these have increased their utilization and production (Bouchenak and Lamri-Senhadji, 2013). Phytochemicals are biologically active compounds that possess health benefits and protect against numerous disorders like diabetes, coronary heart disease, high blood pressure, inflammation, cancer, viral infection and others (Sharma et al., 2011). They advance good health and promote wellness by blocking or treating diseases which are achieved through exerting anti-inflammatory, anti-aging, antioxidant, hypolipidemic, hepatoprotective, anticancer, antiviral, antihypertensive, antidiabetic effects, or by preventing osteoporosis, heart disease, DNA damage and other chronic illness (Oboh, 2006; Prakash and Gupta 2011; Sharma et al., 2011) Many studies have demonstrated that consistent intake of legumes and whole-grains decrease the development of chronic disorders linked with oxidative damage because of the rich phenolic phytochemical contents (Amic et al., 2003; Marathe et al., 2011). Acknowledging the significance role of legume in human nutrition, the World Health Organization (WHO, 1990) recommended at least 30 g of legumes and seeds per day for optimum health and chronic disease prevention.

*Vigna subterraenea* popularly known as Bambara groundnut (BGN) is an underutilized leguminous crop normally utilised as food and for its medicinal benefit in many African countries. Much indigenous knowledge (IK) associate BGN with some medicinal values. Various fractions of BGN plant like the seeds, roots and leaves are used to cure many diseases. For example in South Africa, pregnant women chew and swallow immature fresh seeds to prevent nausea and vomiting; as a treatment for morning sickness during pregnancy (Swanevelder, 1995; DPP, 2011). The black seeds are chewed to alleviate swollen jaw diseases in Ghana, while the milled seeds mixed with water are utilized in the treatment of skin rashes and sick children, the cream coloured BGN seeds are used for treating diarrhea after mixing it with the meat of guinea fowl (Karikari et al., 1995; Ngugi, 1995; Swanevelder, 1995, 1998). Various medicinal contribution of BGN in many African countries has also been reported (Koné et al., 2011). BGN contain high dietary fibre, and its soluble fiber is higher than that of other beans (Koné et al., 2011; Maphosa and Jideani, 2016).

Recently, many phytochemicals with potential nutraceutical application have been reported in BGN. Most of the phytochemicals are phenolics such as flavonoids (quercetin, kaempferol, catechin, epicatechin, medioresinol, catechin dimer anthocyanin) and phenolic acids (chlorogenic acid, p-coumaric acid, ellagic acid, quinic acid, caffeic acid and its derivatives, gallic acid, salicylic acid and *t*-ferulic acid) (Onyilagha et al., 2009; Nyau et al., 2015a; Harris et al., 2018). These BGN components are polyphenolic compounds with different health-promoting properties. Many population-based research have revealed beneficial correlation between intake of polyphenol-rich food and reduction in various non-communicable diseases. For instance, polyphenol in legumes and nuts are known to decrease the possibility or chances of one having diabetes, stroke and ischemic heart disease (Afshin et al., 2014), intake of polyphenol-rich cocoa and chocolate was linked with a decrease in the possibility of developing myocardial infarction and cardiometabolic health (Larsson et al., 2016; Lin et al., 2016), apples rich in flavonoids has been demonstrated to improve endothelial function in people at risk of cardiovascular disease (Bondonno et al., 2018; Fraga et al., 2019). A positive relationship was shown between the consumption of total polyphenol-rich food and cognitive factors (verbal memory and language) in midlife adults. Consistent dietary intake of polyphenol-rich chocolate was shown to decrease the risk of cognitive decline (Kesse-Guyot et al., 2012; Crichton et al., 2016; Moreira et al., 2016). Recently a statistically significant relationship was reported between total polyphenol consumption and the risk of developing breast cancer among postmenopausal women and a reverse relationship between taking in of phenolic acid and postmenopausal breast cancer (Gardeazabal et al., 2019). Due to the fact that the phenolic sets in polyphenols can receive electron with formation of steady phenoxy radical, polyphenols disturb or disorganise series of oxidation reactions in constituents of cells and this is responsible for their ability to prevent various degenerative diseases including different cancer types (Manach et al., 2004; Scalbert et al., 2005). Numerous epidemiologic investigations have revealed that consuming high quantity of leguminous crops is correlated with reduction in the occurrence of various cancer types and of importance is colorectal cancer (CRC) (Hu et al., 1991; Singh and Fraser, 1998; Aune et al., 2009; Wang et al., 2013; Zhu et al., 2015; Gardeazabal et al., 2019).

CRC which is the third most common cancer in men and second in female, is among the prevalent cancer worldwide. In 2018, over one million eight hundred (1.8 million) new occurrence and eight hundred and eighty one thousand (881,000) death from CRC was predicted (Bray et al., 2018). CRC is considered as the cause of one death in every ten cancer cases/death, the mortality rate is second after lung cancer and third in terms of incidence/occurrence (Bray et al., 2018). Regular chemotherapy and radiotherapy treatment for CRC besides having a low cure rate also have side effects. This has resulted in the search for natural alternatives from plant-based foods that are rich in phytochemicals because of their safety, low toxicity and universal acceptability (Fresco et al., 2006). The chemopreventive and therapeutic potentials of several legumes such as soybean, beans, lentil, chickpea, and peanut against CRC have been investigated (Yeh et al., 2006; Yan et al., 2010; Wang et al., 2013; Zhu et al., 2015). Few studies have evaluated the antioxidant properties of BGN, but there is limited or no information on the antiproliferative potentials of BGN fraction against CRC. Therefore this research was undertaken to study the polyphenolics contents and antioxidant capability of anatomical parts and whole seeds of BGN grown in the Mpumalanga province of South Africa and determine their potential cytotoxic and antiproliferative effects against Caco-2 and HT-29 colon cancer.

## 2 Materials and methods

### 2.1 Sample collection

Bambara groundnut seeds used in the study were grown by a farmer in Ehlanzeni district, in Mpumalanga Province, South Africa, between October and early December, 2016 and were harvested in June 2017 using groundnut harvester. The pods were shelled with modified groundnut Sheller and packaged in sealed containers and transported to Cape Peninsula University of Technology (CPUT) for the experiment. The seeds were sorted into black, red and brown BGN according to the different seed colours, kept in airtight plastic containers and stored at 4 °C.

### 2.2 Chemicals

Analytical grade reagents were used for the research. They were utilised the way they were bought from the manufacturer, no further purification was done. Purchased from Zigma-Aldrich were Gallic acid, Folin-Ciocalteu reagent, sodium carbonate, 2, 2 –azobis (2-methylpropionamidine) dihydrochloride (AAPH), 2,2- azino-bis (3-ethylbenzothiazoline-6-sulfonic acid) (ABTS), potassium persulfate, acetate buffer, tripyridyl triazine (TPTZ), iron (111) chloride hexahydrate, ascorbic acid, 4-(Dimethylamino)-cinnamaldehyde) (DMACA), catechin, sodium acetate, quercetin, aluminium chloride, fluorescein, ethanol, and acetone (Zigma-Aldrich GmbH, Sternheim, Germany). 6-hydroxy-2, 5, 7, 8-tetramethylchrman-2-carboxylic acid (Trolox) was obtained from Roche (Roche Diagnostics, Mannheim, Germany). Dimethyl sulfoxide (DMSO), penicillin and streptomycin, sterile phosphate buffer, trypsin and Dulbecco's Modified Eagle's Medium (DMEM) were bought from Lonza (Lonza Group Ltd., Verviers, Belgium). Fetal bovine serum (FBS) was purchased from Gibco (Gibco Life Technologies Corporation, Paisley, United Kingdom) and the flask for tissue culture and 96-well flat-bottom transparent microplates (Greiner). 4-[3-(4-Iodophenyl)-2-(4-nitrophenyl)-2H-5-tetrazolio]-1, 3-benzene disulfonate (WST-1) tetrazolium dye was bought from Roche (Roche Diagnostics, Mannheim, Germany).

### 2.3 Preparation of BGN seeds

Black, red and brown BGN seeds washed with distilled water were dried at 50 °C for 48 h using industrial hot air drier (Geiger and Klotzebucher, Cape Town, South Africa). Whole BGN seeds (black, red and brown) were separately pulverized using hammer mill (PertenMill, Perten Instruments AB, Sweden), sieved (250 mm sieve size) and packaged in high-density polyethylene bags. Bambara groundnut cotyledon and seed coats were produced by first coarse milling of the BGN seeds (black, red and brown varieties) with a Corona manual grain crusher for ease of removing the seed coat manually to produce BGN cotyledon. The cotyledons and their respective seed coats were pulverized with PertenMill (PertenMill, Perten Instruments AB, Sweden), sieved (250 mm sieve size) and packaged in high-density polyethylene bags. The flours produced from black, red and brown BGN were stored at 4–6 °C until required for further analysis.

### 2.4 Preparation of 70% ethanolic BGN extracts

The milled Bambara groundnut whole seeds, cotyledons and seed coats were extracted with a 70% (v/v) ethanol solution using an Ultrasound–Assisted Extraction (UAE) as described by Harris et al. (2018). Briefly, 15 g of BGN flour from whole black, red and brown seeds and those of their anatomical parts (cotyledon and seed coats) were extracted with 150 mL of 70% (v/v) ethanol. The samples were sonicated for 30 min at 25 °C using an ultrasound bath (Lasec SA 2510 Branson ultrasound bath 42 kHz ±6%, USA). The extracted mixture was subjected to centrifugation (15316 x g for 15 min at 4 °C) with Beckman centrifuge (Beckman Coulter Avanti J-E centrifuge, USA) and filtered through Whatman filter paper (0.45 μm pore size). Rotary evaporator (Buchi RE 011 model, Switzerland) was used for reducing/condensing the resulting supernatant to 30 mL under pressure at 40 °C to remove residual ethanol. The various BGN extracts were freeze-dried to get lyophilized power (Bench Top Pro Omnitronics freeze dryer, United Scientific, Germany) after freezing at -80 °C. The resulting freeze-dried extracts were used for the following experiments.

### 2.5 Analysis

#### 2.5.1 Determination of total polyphenol

Folin-Ciocalteu procedure reported by Wolfe et al. (2003) with little modification was used for determination of the total polyphenol composition of the BGN samples. Folin-Ciocalteu assay is taken as an electron transfer antioxidant capability and reducing potential test of a sample. For the analysis, extract of BGN (1.0 mg/mL) was combined with 5 ml of Folin-Ciocalteu reagent (10%) with 4 ml of 75% w/v sodium carbonate. The combination was vortexed for 15 s and sent for incubation (40 °C for 30 min) for development of characteristic blue colour. With the aid of a spectrophotometer (ANALYTIK JENA 200–2004 spectrophotometer, Germany), the absorbance of the extract was estimated at 765 nm. Total phenolic composition of BGN sample was calculated based on a standard curve prepared using 0–250 mg/L of gallic acid. The outcome was given as mg of gallic acid equivalent of sample (GAE)/g.

#### 2.5.2 Determination of total flavanol and flavonol

Total flavonol composition was estimated using the method of Yermakov et al. (1987) as described by Kumaran and Karunakaran (2007). In brief, 2.0 mL (20 g/L) of aluminium trichloride and 3.0 mL of sodium acetate solution (50 g/L) was combined with 2.0 mL of BGN extract (1 mg/mL). The mixture was allowed to incubate for 2.5 h at 20 °C, after which the absorbance was taken at 440 nm using a spectrophotometer. Calculation of the total flavonol was achieved with the aid of standard curve obtained from various concentration of quercetin (20–80 mg/L). The result was communicated as milligram of quercetin equivalent per gram sample (QE)/g. Colorimetric procedure was applied in the estimation of the total flavanol of BGN extract. This was effected colorimetrically at 640 nm using the 4-(Dimethylamino)-cinnamaldehyde (DMACA) method of Delcour and Varebkke (1985). The result obtained was communicated as milligram catechin equivalents per gram sample (CE)/g.

#### 2.5.3 Oxygen radical absorbance capacity (ORAC) assay

Oxygen radical absorbance capacity (ORAC) assay evaluates the antioxidant capability of the BGN extract and this was carried out using the procedure of Ou et al. (2001), as modified by Prior et al. (2003). In brief, 12 μL of BGN extract was mixed with 138 μL of fluorescein (μM) which serve as attack target for free radical on a 96 well microplate. The reaction was started by adding 50 μL 2, 2,-azobis (2-methylpropionamidine) dihydrochloride (AAPH) (768 μM). The fluorescence (emission 538 nm, excitation 485 nm) was noted every I min for 2 h using a Fluorescence plate reader (Thermo Fisher Scientific, Walthan, Mass, USA). Standard used for the assay was Trolox and outcomes were communicated as micro mole per gram sample (μmol/g).

#### 2.5.4 Trolox equivalent antioxidant capacity (TEAC)

The radical cation decolourization test that use the principle of 2, 2′-azinobis (3-ethylbenzothiazolie-6-sulfonate) diammonium salt (ABTS) according to the method of Re et al. (1999) was used for the evaluation of TEAC of the BGN extracts. By combing 8 mM ABTS (in water) and 3mM (final concentration) of potassium persulfate and standing in the dark for 16 h, ABTS∗ was produced. This was adjusted to give an absorbance of 0.700 units at 734 nm. In a 96-well microplate, diluted extract of BGN (25 μL) was combined with 250 μL ABTS∗ solution. The plate was incubated for 30 min at room temperature after which it was read at 734 nm using a Multiskan Spectrum plate reader (Thermo Fisher Scientific, USA). The standard used for the analysis was Trolox and outcomes were communicated as micro mole Trolox Equivalent per gram sample (TE)/g.

#### 2.5.5 Determination of ferric reducing antioxidant property (FRAP)

The procedure reported by Benzie and Strain (1996) was used for FRAP analysis. First, FRAP reagent was prepared by combining acetate buffer (300 mM, pH 3.6), tripyridyl triazine (TPTZ) (10 mM in 40 mM HCl) and 20 mM of FeCl_3_.6H_2_O in the ratio 10: 1: 1 (v/v/v). Then, in a 96 well plate, 10 μL of BGN extracts (diluted) were combined with 300 μL of the FRAP reagent and allowed to incubate for 30 min at room temperature. The plate was read after incubation at 593 nm (wavelength) using Multiskan Spectrum plate reader (Thermo Fisher Scientific, USA). The standard used for the assay was Ascorbic acid. All determination was carried out in triplicates.

#### 2.5.6 Evaluation of the effect of BGN extracts on colon cancer cells

##### 2.5.6.1 Cell culture and maintenance

Human colon adeno carcinoma: HT-29 and Caco-2 colon cancer cell lines used for the study were from America Type Culture Collection (ATCC). Dulbecco's Modified Eagle's Medium (DMEM, Lonza Group Ltd., Verviers, Belgium) that was added 10% foetal bovine serum (FBS Gibco Life Technologies Corporation, UK), 1% penicillin and 50 mg/mL of streptomycin (Lonza Group, Ltd) was used for growing the cells. The cell cultures were sent for incubation in an incubator (humidified air) at 37 °C and 5% CO_2_ saturation, with change of medium at three days interval. Cells on attaining 70–80% confluency were sub-cultured using trypsin solution (Lonza) for further experiment.

##### 2.5.6.2 WST-1 cell viability assay

The in vitro analysis, WST-1 (4-[3-(4-iodophenyl)-2-(4-nitrophenyl)-2H-5-tetrazolio]-1,3-benzene disulfonate) was used to measure the cell viability or cytotoxicity of the BGN extracts. The test was carried our using the procedure reported by Ngamwongsatit et al. (2008). Seeding of HT-29 and Caco-2 cancer cells was done in 100 μL DMEM medium on 96 well plate, the cell density was 1 x 10^5^ in each well and was cultured at 37 °C for 24 h for the cells to attach. At the end of 24 h incubation, attached cells were treated by replacing the culture medium with different concentrations (7.5, 15, 30, 60 mg/mL) of BGN extracts and incubated for another 24 h. For the positive control, 6% DMSO was used on the cells, while wells for the blank have equal amounts of growth medium without cells. At the end of 24 h, the remaining media was removed and to each well, 100 μL new/fresh medium and 10 μL of WST-1 (tetrazolium dye) was added and sent for incubation at 37 °C for 4 h. After 4 h, the absorbance was read at 440 and 630 nm using BMG Labtech Omega POLAR Star multimodal plate reader (BMG Labtech, Germany). The WST-1 assay estimates the capability of viable cells with oxidoreductase enzymes that depend on Nicotinamide adenine dinucleotide phosphate (NADPH), to reduce the WST-1 tetrazolium dye to formazan. The percentage cell viability was estimated as the percentage mean absorbance ratio of treated cells against that of untreated cells multiplied by 100. That is Cell viability (%) = (OD440 treated sample/OD440 Untreated cells) ×100. A survival curve was constructed and IC_50_ values determined using Graph Pad Prism 6 software (Graph Pad software, San Diego, CA, USA).

### 2.6 Statistical analysis

Data were represented as mean ± SEM of three independent replicates. GraphPad Prism software (GraphPad Prism software, San Diego, CA, USA) was used to perform the statistical analysis, while Multivariate analysis of variance was used to determine difference between treatment means. Duncan multiple range test was used for mean separation (IBM-SPSS, 15) and significant difference was determined at p < 0.05. The IC_50_ values were generated from the WST-1 results using GraphPad Prism**.**

## 3 Results

### 3.1 Total polyphenol, flavonol and flavanol content of BGN varieties and fractions

Total phenolic, flavonol, and flavanol contents of whole black, red and brown BGN landraces, their cotyledons and seed coats are presented in Table 1. There was a significant difference (p < 0.05) in total polyphenol content among the BGN varieties and the fractional parts. Bambara groundnut seed coats had significantly (p < 0.05) more total polyphenol content then the whole seeds and cotyledon. The brown BGN landrace had the highest total polyphenolic content, which was significantly (p < 0.05) higher than that of the whole seed and the cotyledon. A similar trend was observed in black and red landraces. However, compared to other varieties, the black BGN landrace had the lowest content of polyphenols in the whole seed and fractional parts. In all the varieties, the cotyledon had the lowest polyphenolic content. Similarly, flavonoid content (flavanol and flavonol) of the seed coats of the various BGN landraces were significantly (p < 0.05) higher than that of the whole seeds and cotyledon. However, flavonol content of the black BGN whole seed was comparable to the seed coat content of the brown variety. Among the samples, flavanol content of the seed coats of the brown BGN landrace was significantly (p < 0.05) more than the red and black ones. The red BGN cotyledon had the lowest flavanol content. Flavanol was not detected in the cotyledon of the black and brown landraces.

**Table 1:** Total phenolic, flavonol, and flavanol contents of black, red and brown BGN landraces and their cotyledons and seed coats.

BGN Types/parts	Polyphenol (mg/g)	Flavonol (mg/g)	Flavanol (mg/g)
Whole			
Black	5.39 ± 0.13^a^	7.04 ± 0.59^c^	2.26 ± 0.03^a^
Red	8.30 ± 0.38^a,c^	4.15 ± 0.24^b^	2.39 ± 0.13^a^
Brown	10.30 ± 0.52^c^	5.08 ± 0.26^b^	3.87 ± 0.11^c^
Cotyledon			
Black	3.01 ± 0.22^g^	1.21 ± 0.05^a^	N.D
Red	4.48 ± 0.15^h^	7.12 ± 0.46^c^	1.65 ± 0.09^h^
Brown	3.28 ± 0.14^g^	5.05 ± 0.30^b^	N.D
Seed coat			
Black	153.41 ± 6.45^f^	9.09 ± 1.07^d^	37.08 ± 1.02^d^
Red	213.76 ± 6.48^e^	9.98 ± 0.52^d^	62.25 ± 0.51^e^
Brown	288.08 ± 5.23^d^	7.28 ± 0.57^c^	101.77 ± 1.90^f^

Values are mean ± SEM for three replicates. Means in the same column followed by different superscript letters are significantly (p < 0.05) different. N.D – detected.

### 3.2 Antioxidant properties of BGN varieties and their fractions

Antioxidant activity of the BGN varieties and their fractions as determined with the ABTS, ORAC, and FRAP assays are shown in Table 2. The outcome indicated that the seed coat had significantly (p < 0.05) greater antioxidant activity than the whole seed or the cotyledon. ORAC and ABTS antioxidant activity of the seed coats were significantly (p < 0.05) more than that of the whole seeds and cotyledons. Similarly, FRAP antioxidant activity of the various BGN seed coat was also significantly (p < 0.05) higher than whole BGN seed and cotyledon. Generally, the cotyledon had significantly (p < 0.05) lower antioxidant activity with the cotyledon of the black BGN landrace having the lowest antioxidant activity.

**Table 2:** Total antioxidant activity- ORAC, ABTS and FRAP black, red and brown BGN landraces, their cotyledons and seed coats.

BGN Types/parts	ORAC (μmol TE/g)	ABTS/(μmol TE/g)	FRAP (mg/g)
Whole			
Black	343.62 ± 16.09^b^	40.26 ± 0.75^a^	14.21 ± 0.51^a^
Red	525.42 ± 14.34^c^	67.70 ± 1.031^b^	24.43 ± 3.04^b^
Brown	405.69 ± 6.42^e^	86.13 ± 0.9^c^	36.30 ± 1.55^c^
Cotyledon			
Black.	151.13 ± 1.47^a^	40.05 ± 4.13^a^	2.95 ± 0.32^d^
Red	361.20 ± 11.46^b^	38.68 ± 1.06^f^	11.67 ± 0.24^e^
Brown	209.53 ± 2.59^f^	33.42 ± .0.09^z^	8.82 ± 0.46^f^
Seed coat			
Black	2521.78 ± 150.51^g^	1104.04 ± 99.59^g^	636.84 ± 38.44^g^
Red	3147.44 ± 168.46^h^	1412.37 ± 37.63^h^	1025.9 ± 5.37^h^
Brown	4181.90 ± 18.99^k^	1452.47 ± 0.54^k^	1244.4 ± 52.11^j^

Values are mean ± SEM for three replicates. Means in the same column followed by different superscript letters are significantly (p < 0.05) different.

### 3.3 Cytotoxicity and antiproliferative activity of BGN extracts on Caco-2 and HT-29 cancer cells

**Figure 1:**
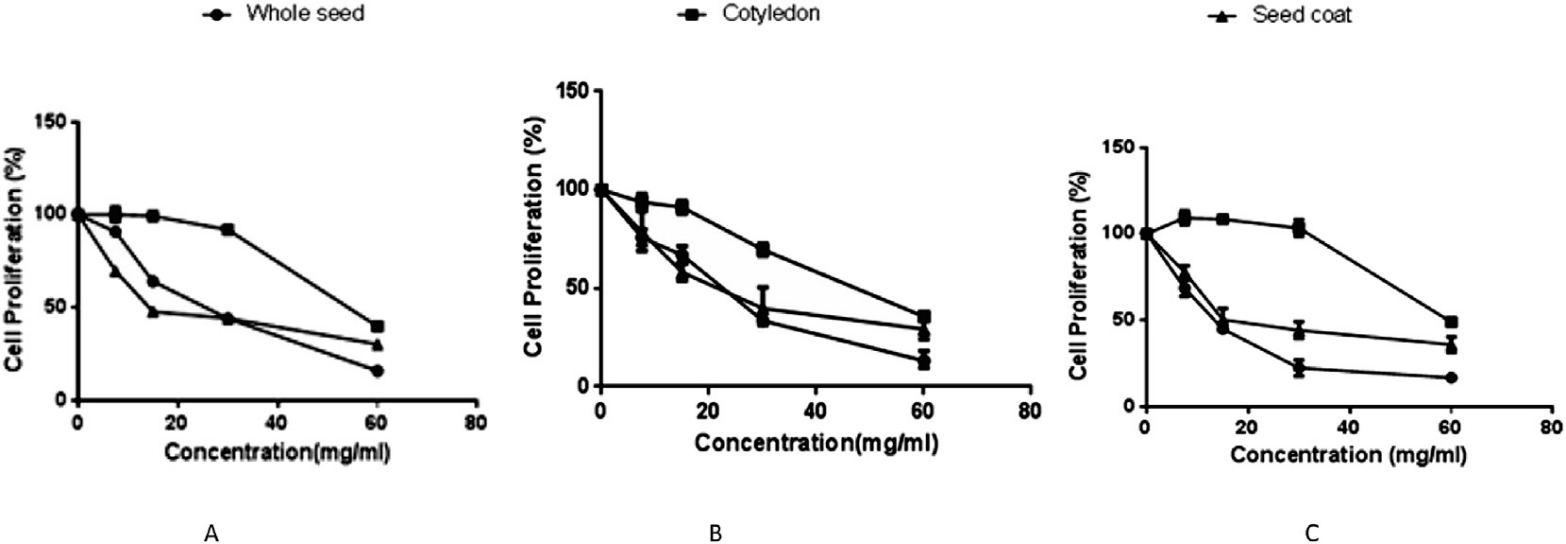
Effect of different concentrations of 70% ethanolic extracts from black, red and brown BGN whole seeds, cotyledons and seed coats on CaCo-2 colon cancer cell proliferation. A. Effect of different concentrations of 70% ethanolic extracts from black BGN whole seed, cotyledon and seed coat on CaCo-2 colon cancer cell proliferation. B. Effect of different concentrations of 70% ethanolic extracts from red BGN whole seed, cotyledon and seed coat on CaCo-2 colon cancer cell proliferation. C. Effect of different concentrations of 70% ethanolic extracts from brown BGN whole seed, cotyledon and seed coat on CaCo-2 colon cancer cell proliferation.

**Figure 2:**
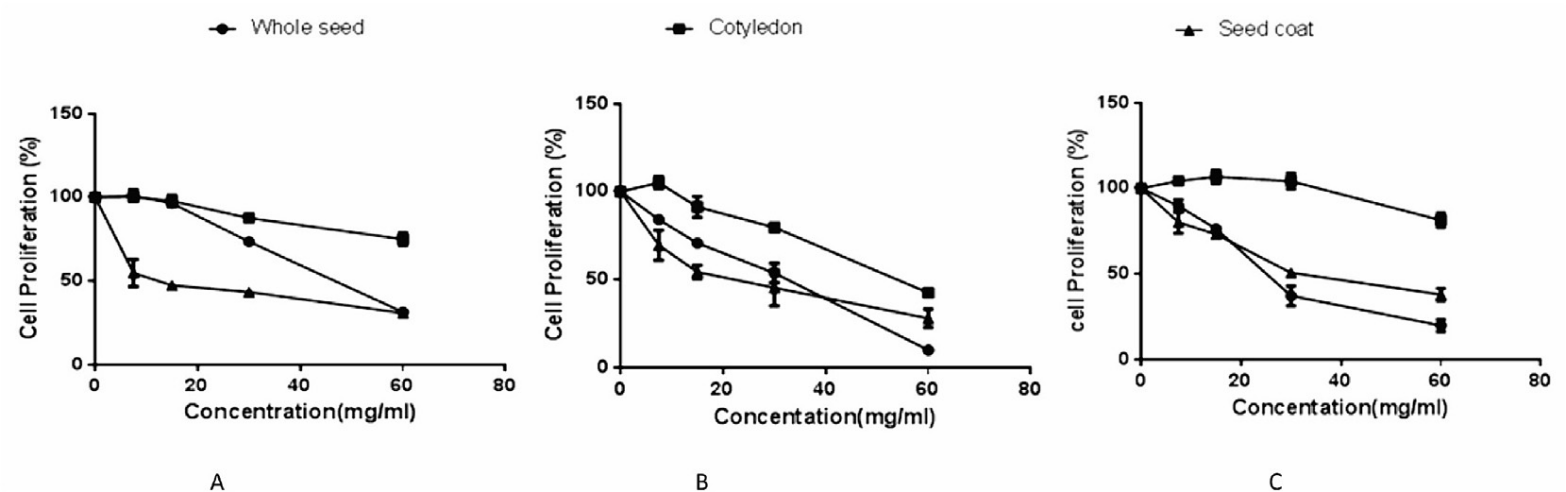
Effect of different concentrations of 70% ethanolic extracts from black, red and brown BGN whole seeds, cotyledons and seed coats on HT-29 colon cancer cell proliferation. A. Effect of different concentrations of 70% ethanolic extracts from black BGN whole seed, cotyledon and seed coat on HT-29 colon cancer cell proliferation. B. Effect of different concentrations of 70% ethanolic extracts from red BGN whole seed, cotyledon and seed coat on HT-29 colon cancer cell proliferation. C. Effect of different concentrations of 70% ethanolic extracts from brown BGN whole seed, cotyledon and seed coat on HT-29 colon cancer cell proliferation.

**Figure 3:**
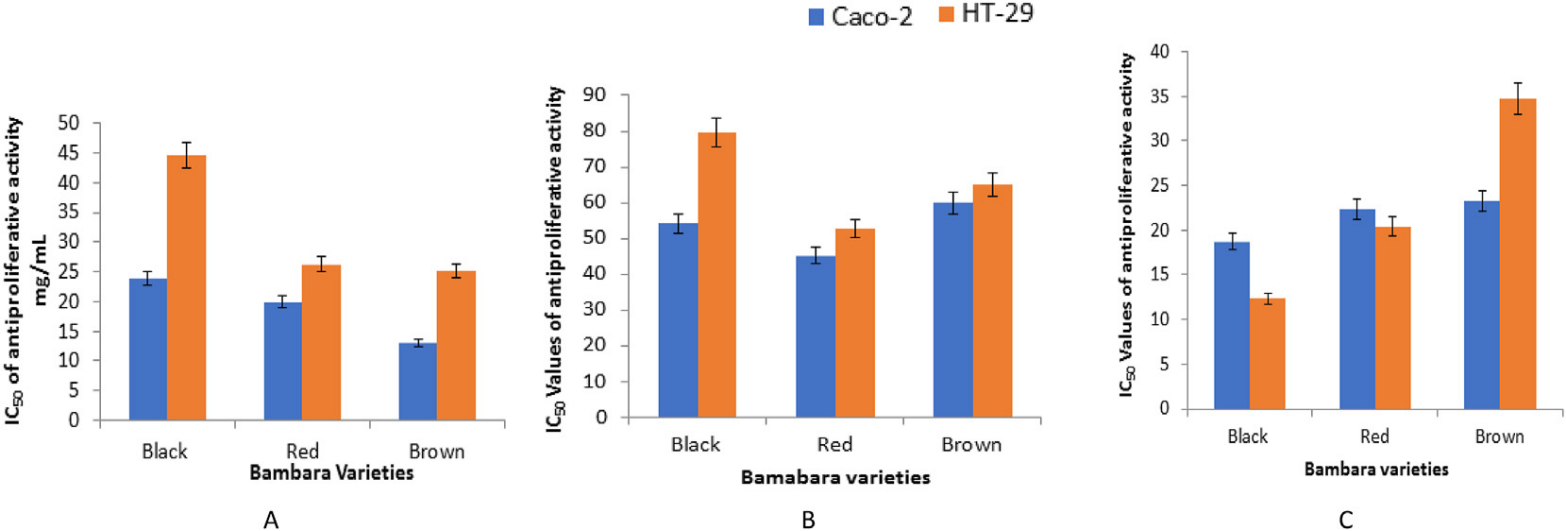
IC_50_ values of antiproliferative activity of 70% ethanol extracts of whole seeds of black, red and brown BGN varieties on Caco-2 and HT-29 (mean ± SEM, n = 3). A. IC_50_ values of antiproliferative activity of 70% ethanol extracts of whole seeds of black, red and brown BGN varieties on Caco-2 and HT-29 (mean ± SEM, n = 3). B. IC_50_ values of antiproliferative activity of 70% ethanol extracts of the cotyledons of black, red and brown BGN varieties on Caco-2 and HT-29 (mean ± SEM, n = 3). C. IC_50_ values of antiproliferative activity of 70% ethanol extracts of seed coats of black, red and brown BGN varieties on Caco-2 and HT-29 (mean ± SEM, n = 3).

The cytotoxicity and antiproliferative activities of the three varieties of BGN and their anatomical parts towards the growth of HT-29 and Caco-2 colon cancer cells in an *in vitro* experiment are presented in Figures 1A, B, C and 2A, B and C, respectively. Among the BGN parts, the ethanolic extracts of the whole seed and seed coat revealed significantly (p < 0.05) higher antiproliferative activities towards both Caco-2 and HT-29 cells in a dose-dependent manner. The calculation of the halfway point that a particular compound achieves total or complete inhibition of a biochemical or biological function is known as IC50, so the antiproliferative potentials of the BGN fractions are communicated as the half-maximal inhibitory concentration (IC_50_). In this study, a higher IC_50_ value indicate lower antiproriferative capability while lower IC_50_ values shows higher effect/cancer cell inhibition (Figure 3A, B and C). Caco-2 cancer cells proliferation were significantly (p < 0.05) inhibited according to dosage of ethanolic extracts of black, red and brown BGN varieties administered (Figure 1A, B and C). The seed coat and the whole seed showed significantly (p < 0.05) higher cytotoxic and antiproliferative activity and Caco-2 cancer cell inhibition compared to the cotyledon. The ethanolic extracts of brown, red and black BGN whole seed (13.07, 19.95 and 23.89 mg/mL) and seed coat (23.29, 22.37 and 18.692 mg/mL) exhibited the highest antiproliferative and cancer cell inhibition towards Caco-2 cancer cell and had lower IC_50_ values (Figure 3A and C). Extracts of the cotyledon exhibited the lowest antiproliferative activity and cancer cell inhibition toward Caco-2 cells at higher doses, with IC_50_ of 59.9, 45.1 and 54, 23 mg/mL, respectively (Figure 3B). Similarly, HT-29 colon cancer cell proliferation was significantly (p < 0.05) inhibited according to dosage of ethanolic extracts of black, red and brown BGN varieties administered. The ethanolic extracts of whole BGN seeds and seed coats of brown, red and black demonstrated the highest antiproliferative effect (p < 0.05) toward HT-29 colon cancer cells with the lowest IC_50_ of 25.16, 26.23 and 44.59 mg/mL (whole seeds) and 34.74, 20.41 and 12.38 mg/mL (seed coat), respectively (Figures 2A, B, C and 3A, C). The ethanolic extract of BGN cotyledon exhibited weaker or lower antiproliferative activity and HT-29 colon cancer cell inhibition at higher doses with IC_50_ values 65.17, 52.76 and 79.5 mg/mL, respectively (Figures 2A, B, C and 3B). Generally, 70% ethanolic BGN extracts exhibited greater cytotoxicity and antiproliferative activity towards Caco-2 colon cancer cell than HT-29 colon cancer cells.

## 4 Discussion

Plant phenolics are common components of food crops such as legumes, vegetables, fruits, cereals and others. They include flavonoids, phenolic acids, tannins, stilbenes and lignans. Among these, flavonoids are the most abundant in our foods and they are subdivided into six classes that include: Flavonols, flavanols (flavan-3-ol), flavones, isoflavones, flavanones and anthocyanins, based on the central C ring's oxidation state. Various classes of phenolic compounds have been reported in BGN including, flavonoids (quercetin,quercetin-3-O-rutinoside, quercetin -3-O-glucoside, catechin, myricetin, rutin and kaempferol, epicatechin, medioresinol, catechin and medioresinol), while chlorogenic acid, quinic acid, gallic acid, ellagic acid, *t-*feruric acid, salicylic acid, caffeic acid and its derivatives and p-coumaric acid were the phenolic acids present (Onyilagha et al., 2009; Nyau et al., 2015a; Harris et al., 2018).

In this research extraction of phenolic compounds was carried out using aqueous ethanol (polar mixture) due to the fact that this type of protic polar solvent is preferred and better for the initial stage in the series of processes involved in extracting phenolics, and have been used and successfully employed in extraction of glycosides of flavonoids and other phenols that the molecular weight is high and with increased yield (Oreopoulou and Tzia, 2007; Akowuah et al., 2005; Tsakona et al., 2012; Galanakis et al., 2013). This mixture of solvent compared to ethanol and ethyl acetate have also been shown to extract greatest quantity of various class of phenols (Obied et al., 2005). The presence of water in aqueous alcohol mixture during extraction causes the plant components to absorb water and expand, this allows the extracting solvent to enter and interact easily and very well with the solid matrix, thus resulting in greater extraction of bioactive components of the plant, this explains why aqueous-alcohol mixture/solvent seem better than other conventional or normal solvents in extracting phenolic compounds (Galanakis et al., 2013). In this study, polyphenol and flavonoid (flavanol and flavonol) were present in the various BGN parts but in higher quantity in the seed coat than in the whole seed and cotyledon, seed coat of brown BGN has the highest phenolic content. This is consistent with the findings that reported higher concentrations of phenolics like tannins and flavonoids in red and brown BGN seed coat compared to whole seed and cotyledon (Harris et al., 2018). Similarly, high total polyphenolic content of 411.16 mg GAE in the BGN seed coat has been reported (Klompong and Benjakul, 2015). However, the higher total polyphenol content in the study of Klompong and Benjakul (2015) compared to our experiment could probably be due to different extraction method used and also variance in soil/other environmental conditions. The reason why seed coat has higher total polyphenol content than whole seed and cotyledon is because of uneven distribution of phenolics in these parts, they are more concentrated in the outer layer (seed coat) of legumes (Dueñas et al., 2006). Natural antioxidants and polyphenols could be derived from legume seed coats (Moise et al., 2005; Dueñas et al., 2006). Therefore, processing treatments such as dehulling results in the removal of the phenolic compounds thereby reduction in the overall polyphenol content of processed legumes which includes BGN (Duodu, 2014).

Plant polyphenols has gained increase research attention due to their strong antioxidant potentials, and this has been applied in the prevention/inhibition of many diseases associated with oxidative stress like cancer. Polyphenols has been shown to be more potent than carotenoids, vitamin E and C in various in vitro experiments because of its strong antioxidant activity (Rice-Evans et al., 1995; Rice- Evans et al., 1996). Antioxidants are substances that are capable of delaying, preventing or inhibiting materials capable of oxidation from been oxidized by seeking free radicals and decreasing stress caused by oxidation (Cotelle, 2001). Phenolic substances achieve their antioxidant properties by scavenging radical nitrogen and radical oxygen species (RNS and ROS), inhibiting numerous enzymes and chelating trace metals taking part in the production of free radicals by suppression of ROS and RNS formation, and increasing the rate and protection of antioxidant defence (Cotelle, 2001).

ORAC antioxidant activity is an assay that measures the ability of substances that prevent oxidation (antioxidants) to stop free radicals activity by donating hydrogen atom (Cao et al., 1995). In this investigation, BGN seed coat, irrespective of the variety, exhibited strong ORAC antioxidant properties which were significantly (p < 0.05) higher than that of the whole seed and cotyledon. The ORAC antioxidant activity of the BGN seed coat, whole seed and cotyledon could be linked with the total polyphenols content as they follow the same trend. The higher content of total polyphenols in BGN seed coats reflects their higher antioxidant activity. This finding is in agreement with other reports that revealed that the phenolic composition of plants is associated with its antioxidant property or the ability to scavenge free radicals (Ademiluyi and Oboh, 2008; Chu et al., 2002). The strong free scavenging ability and potentials of the seed coat of different BGN landraces definitely show the high content of phenolics. BGN cotyledon had lowest ORAC antioxidant activity irrespective of the BGN variety.

In the present investigation, ABTS scavenging activity of the seed coat of the three BGN landraces was significantly (p < 0.05) greater than the one for cotyledon and whole BGN seed. It was reported that seed coats of BGN extracted at various temperatures with water, acetone and ethanol had high ABTS radical scavenging activity (Klompong and Benjakul, 2015) and the values were similar to the ABTS activity obtained in this study. They also demonstrated that the ability of ABTS to scavenge for free radicals is associated with the overall phenolics present (Klompong and Benjakul, 2015). Similarly, the seed coats of brown and red BGN varieties have been shown to have greatest accumulation of tannins and flavonoids in contrast to the cotyledon and whole seeds (Harris et al., 2018). It was also reported that phenolics like tannins whose molecular weight is high have greater power to extinguish unbound or loose radicals (ABTS+) and that the efficacy is determined not by the specific functional group but by other factors like the aromatic ring number, molecular mass and interchange of the hydroxyl group (Hagerman et al., 1998; Siddhuraju and Becker, 2007).

The strong scavenging activity of BGN seed coats extracts against ABTS radical could probably be due to their ability to donate electrons to the ABTS radical.

The FRAP assay of the BGN extracts evaluated its potential to decrease Fe^3+^ - TPTZ complex to Fe^2+^ which is the ferrous form, denoting antioxidant capacity requiring transfer of a single electron. It was demonstrated that the FRAP antioxidant activity follows the same trend as the total phenolic content. FRAP antioxidant capability of the seed coat of brown BGN was higher than that of the whole seed while that of the cotyledon was the lowest. The FRAP antioxidant ability obtained in this experiment is higher than those reported for ethanol extracted BGN seed coats (Klompong and Benjakul, 2015). The difference in the FRAP values could be due to varietal, environmental difference and extraction solvent. However, the FRAP activity was comparable to those of BGN whole seeds reported by Nyau et al. (2015b) and the USA market class lentils. However, the FRAP values were greater than those of chickpea, yellow peas, common beans and green peas cultivated for the USA market in North Dakota, Washington and Idaho regions (Xu et al., 2007). The relatively high FRAP capability of extracts of BGN seed coat may be due to higher concentration of phenolics present in the seed coat. It has been demonstrated that the reducing ability of phenolics of low and high molecular mass obtained from Indian laburnum's stem bark and hulls of peanut were associated to their free radical scavenging property (Yen and Duh, 1993; Siddhuraju et al., 2002).

All over the world CRC is a major cause of death resulting from cancer. Studies have shown that 90–95% of cancer cases are caused by lifestyle and environmental factors in which diet plays a very important role (Li et al., 2015). This means that cancer could be prevented by the type of food consumed. This is especially true of colon cancer that shows lengthy precancerous phase that offers the patient chance to hinder or inhibit the development of adenomas into cancer. Research and population based studies have revealed that meals with plenty vegetables, fruits, grains and spices are correlated with decrease in chances of CRC initiation and growth (Steinmetz and Potter, 1996; Greenwald, 2005). Food dietary phytochemical components are mostly responsible for the anticancer properties of these foods.

It has been shown that the strong antioxidant and cancer inhibition potentials of fruit, vegetables and other crops is due to the additive and collective effects of the phytochemicals they contain, and the gain and benefit of the meals with plenty of this substances are attributed to the interactivity or reactions of its phytochemicals (Liu, 2003). These bioactive food components act as chemopreventive agents and in some cases could be chemotherapeutic. When consumed, they restrain cancer development, stop or check its advancement or continuation or bring about reverse in cancer formation at a premalignant phase (Sporn and Suh, 2002). It was observed that exposure of 70 % ethanolic extracts of BGN whole seed, cotyledon and seed coat to colon cancer cells (Caco-2 and HT-29) showed cytotoxicity and inhibited cell proliferation in a dose-dependent way, with the whole seed and seed coat of the different BGN varieties demonstrating greater cytotoxicity and antiproliferative activity than the cotyledon. The antiproliferative activity of the various BGN parts had different effects on HT-29 and Caco-2 cancer cell lines. Generally, the various fractions of BGN showed exceptional capability in inhibiting the proliferation of Caco-2 cancer cells than HT-29.

The cytotoxicity and inhibition of colon cancer proliferation by BGN extract could be partially due to the phenolcs present in various BGN parts which may have worked additively or synergistically to effect the antiproliferative activities. Research has shown that various BGN landraces have phenolics like flavonoids and phenolic acids which include epicatechin, catechin, catechin glucoside, quercetin-3-O-rutinoside, quercetin-3-O-glucoside (flavonoids) while caffeic acid and its derivatives, quinic acid, p-coumaric acid, salicylic acid and *t*-feruric acid are phenolic acids (Nyau et al., 2015a). Phenolic compounds were previously identified in BGN varieties investigated in the present study, they include flavonoids (quercetin, catechin, myricetin, rutin and kaempferol) and phenolic acids (gallic acid, ellagic acid and chlorogenic acids) (Harris et al., 2018). Many of these phenolic substances are known to exhibit chemopreventive and chemotherapeutic properties against colon cancer and the mechanisms of those are well-established. It was shown that Manuka honey phenol-rich extract containing caffeic acid, gallic acid, luteolin, quercetin and kaempferol demonstrated strong chemopreventive outcome on LoVo cells and colon cancer cells at various concentrations (0–50 mg/mL^−1^) (Afrin et al., 2018). The profound inhibitory activity was achieved by a reduction in colon cancer cells proliferation ability, effecting/causing apoptosis and arresting of cell cycle. Rise in the spreading of p53 protein, cleaved-PARP and caspase-3 caused apoptosis and inherent (caspase-9) and external (caspase -8) pathways of apoptosis were triggered and this brought about suppression of p-Akt protein with rise or growth in expressing endoplasmic stress markers like p-Erk1/2, p-p38MARK, XBP1 and ATF6 (Afrin et al., 2018). The extract effected arrest of the cell cycle in HCT-116 cells at the S phase and at G2/M phase in LoVo cells by modifying the cell cycle regulator genes (p21, p27, Bb, cyclin D1, cyclin E, CDK2 and CDK4) (Afrin et al., 2018). Similar findings were reported for honey by various researchers who showed that Gelam honey, Nenas honey and India commercial containing quercetin, luteolin, kaempferol and caffeic acid exhibited antiproliferative and inhibitory effects on HCT-116, HT-29 and HCT-15 colon cancer cells and indicated that these phenolics played a very important part in supressing cancer cell proliferation (Jaganathan and Mandal, 2010; Wen et al., 2012; Tahir et al., 2015; Kee et al., 2016; Afrin et al., 2017). In a recent study on Manuka honey anticancer effect on HCT-116's cancer stem cells (CSCs), the size of culture spheroids was reduced and there was also induction of apoptosis, decreased expression of mRNA of one ABC transporter (ABCG2) and down modulation of mRNA expression of the receptor membranes of Wnt/β-catenin pathway because of the polyphenolic content of the honey extracts (Cianciosi et al., 2020).

Quercetin of 10.5 mmol/L in a study of the inhibitory potential of 12 flavonoids on the transcriptional capability of Cyclooxygenase-2 (COX-2) gene in DLD-1 human colon cancer cells using a reporter gene assay, was shown to suppress the activity of COX-2 transcription most effectively (Mutoh et al., 2000). Similarly, it was reported that HT-29 cancer cells treated with quercetin resulted in significant reduction in cell proliferation and viability, induced arrest of cell cycle in the G1 stage and brought about rise in expression of AMPK, p53 and p21 which are proteins that cause apoptosis (Kim et al., 2010). *In vivo* experiment showed that treatment of colon cancer cells with quercetin for six weeks brought a remarkable decrease in cancerous growth (tumor) size, this indicated that quercetin might have effected apoptosis by activating the AMPK and causing apoptotic death that was dependent on p53 in HT-29 cancer cells. Ellagic acid is a rich phenolic acid present in BGN and it was shown that 15 days administration of ellagic acid against murine colon cancer induced with a carcinogenic compound (1,2-dimethyl hydrazine) resulted in prevention of the activation of P13k/Akt pathway linked with colon carcinogenesis (Umesalma and Sudhandiran, 2011). Research has demonstrated that other phenolic acids found in BGN such as caffeic acid phenyl ester (CAPE) and caffeic acid not only inhibited the invasion of colon cancer cells but equally brought about a reduction in MMP-2/-9 and VEGF production (Liao et al., 2003). Dietary fiber is another important component in BGN that may have caused the inhibition of HT-29 and Caco-2 cancer cell proliferation. BGN was reported to have the highest content of soluble fiber when compared to other beans and is also rich in dietary fiber (Koné et al., 2011; Jideani and Diedericks, 2014). Dietary fiber has been reported to aid in colon cancer chemoprevention by probably increasing stool bulk, reducing transit time and diluting potential carcinogens in the gastrointestinal tract. It also activates bacterial anaerobic fermentation with resultant production fatty acids (short-chains) like butyrate that result in colorectal cancer growth inhibition, apoptosis induction, arrest of cell cycle and encourage differentiation of cells in colon cancer cells (Lipkin et al., 1999). These compounds contained in BGN may have worked individually or synergistically or in combination with other BGN components to effect cytotoxicity and inhibition of HT-29 and Caco-2 colon cancer cells demonstrated in this study. Some of them such as dietary fiber, quercetin and kaempferol have been shown to inhibit colorectal tumour multiplicity in animal studies at low concentrations of 0.3–0.5 % that are relevant for human diets (Bobe et al., 2013).

The phenolic rich extract of BGN parts in this study could also be explored for other uses as they contain phenolics like hydroxyl-cinnamic acid derivatives, flavonoids (flavonols and flavanols) and o-diphenols especially the seed coat (Harris et al., 2018). These class of phenolics extracted from olive cake, olive kernel, olive mill wastewater (Galanakis et al., 2010), have been successfully utilized commercially as biologically active components and preservatives from natural source (Rahmanian et al., 2014; Galanakis, 2017), in the fortification and preservation of various commodities like bakery products, table olives, lard, vegetable oil, milk beverages and meat products (Lalas et al., 2011; Servili et al., 2011b; Chaves-López et al., 2015; Yangui et al., 2015; Balzan et al., 2017; Veneziani et al., 2017; Galanakis et al., 2018a, 2018d; Serra et al., 2017). They have also been successfully used in the formulation of UV booster in cosmetics (Rahmanian et al., 2014; Galanakis et al., 2018b). So BGN rich phenolics extracts could also be explored for these tailored/specific applications after further research.

In conclusion, 70% ethanolic BGN seed coat extract contained relatively greater quantity of polyphenolic components and demonstrated better antioxidant capability, though varying amount exists in the whole seed and cotyledon. In addition, HT-29 and Caco-2 colon cancer cell viability and proliferation were remarkably inhibited in a dose-dependent way after treatment with 70% ethanolic BGN extracts, with seed coat and whole seed extracts exhibiting the highest cytotoxicity and antiproliferative activity and cancer cell inhibition. This result provides new knowledge on the possible health benefits of BGN, as well as the potential for product development. However, additional research is needed for further identification of the BGN bioactive components and specific phenolics and other phytochemicals behind its antioxidant and anticancer potentials. This will guide their possible nutraceutical and pharmaceutical exploitation and application.

## Declarations

### Author contribution statement

Jane N. C. Okafor: Conceived and designed the experiments; Performed the experiments; Analyzed and interpreted the data; Wrote the paper.

Fanie Rautenbauch: Analyzed and interpreted the data; Contributed reagents, materials, analysis tools or data.

Mervin Meyer, Victoria A. Jideani: Conceived and designed the experiments; Analyzed and interpreted the data; Contributed reagents, materials, analysis tools or data.

Marilize Le Roes-Hill: Conceived and designed the experiments; Contributed reagents, materials, analysis tools or data.

Taahir Harris: Performed the experiments.

### Funding statement

This work was supported by the National Research Foundation (NRF) of South Africa.

### Data availability statement

Data included in article/supplementary material/referenced in article.

### Declaration of interests statement

The authors declare no conflict of interest.

### Additional information

No additional information is available for this paper.
